# EphA2 Is a Potential Player of Malignant Cellular Behavior in Non-Metastatic Renal Cell Carcinoma Cells but Not in Metastatic Renal Cell Carcinoma Cells

**DOI:** 10.1371/journal.pone.0130975

**Published:** 2015-07-15

**Authors:** Min Chul Cho, Sung Yong Cho, Cheol Yong Yoon, Seung Bae Lee, Cheol Kwak, Hyeon Hoe Kim, Hyeon Jeong

**Affiliations:** 1 Department of Urology, Dongguk University College of Medicine, Seoul, Korea; 2 Department of Urology, SMG-SNU Boramae Medical Center, Seoul, Korea; 3 Department of Urology, Korea University Guro Hospital, Seoul, Korea; 4 Department of Urology, Seoul National University College of Medicine, Seoul, Korea; Cedars Sinai Medical Center, UNITED STATES

## Abstract

**Objectives:**

To investigate the role of EphA2 in malignant cellular behavior in renal cell carcinoma (RCC) cells and whether FAK/RhoA signaling can act as downstream effectors of EphA2 on RCC cells.

**Methods:**

Expression of EphA2 protein in non-metastatic RCC (Caki-2 and A498), metastatic RCC cells (Caki-1 and ACHN), HEK-293 cells and prostate cancer cells (PC-3 and DU-145; positive controls of EphA2 expression) was evaluated by Western blot. Changes in mRNA or protein expression of EphA2, FAK or membrane-bound RhoA following EphA2, FAK or RhoA small interfering RNA (siRNA) transfection were determined by reverse transcription polymerase chain reaction or Western blot. The effect of siRNA treatment on cellular viability, apoptosis and invasion was analyzed by cell counting kit-8, Annexin-V and modified Matrigel-Boyden assays, respectively.

**Results:**

In all RCC cell lines, the expression of EphA2 protein was detectable at variable levels; however, in HEK-293 cells, EphA2 expression was very low. Treatment with EphA2 siRNA significantly reduced the expression of EphA2 mRNA and protein in all RCC cell lines. For non-metastatic RCC cells (Caki-2 and A498) but not metastatic RCC cells (Caki-1 and ACHN), cellular viability, invasiveness, resistance to apoptosis, expression of membrane-bound RhoA protein and FAK phosphorylation were significantly decreased in EphA2 siRNA-treated cells compared to the control. In non-metastatic RCC cells, FAK siRNA significantly attenuated the invasiveness, resistance to apoptosis, as well as expression of membrane-bound RhoA protein without changing protein expression of EphA2. RhoA siRNA significantly decreased the malignant cellular behavior and expression of membrane-bound RhoA protein without changing EphA2 protein expression or FAK phosphorylation.

**Conclusions:**

Our data provide the first functional evidence that the EphA2/FAK/RhoA signaling pathway plays a critical role in the malignant cellular behavior of RCC and appears to be functional particularly in the early stage of malignant progression of non-metastatic RCC.

## Introduction

Approximately 25% of patients with renal cell carcinoma (RCC) present distant metastases at diagnosis and approximately 30% of RCC patients eventually develop metastases during the disease course [1]. Furthermore, advanced RCC is highly refractory to conventional therapy including radiation and chemotherapy, and the effectiveness of immunotherapy is still controversial [2]. Targeted therapy such as tyrosine kinase inhibitors and mammalian target of rapamycin (mTOR) inhibitors have been introduced recently, but there are no data to indicate that it is curative, and most patients who undergo this therapy eventually relapse, leading to death from RCC [3]. Thus, managing advanced RCC remains one of the most significant challenges to clinicians and underscores the need for development of more effective systemic therapies against disease progression.

Eph, the largest family of receptor tyrosine kinases, is known to play important roles in malignant cellular behavior in many types of tumors [4]. EphA2, a member of the Eph family, is overexpressed in tumor cells of various types of cancer including breast, prostate and colon [5]. Additionally, increased EphA2 expression can promote tumor progression by inducing cancer cell growth and invasion while concurrently decreasing apoptosis [5]. A previous study showed that higher levels of EphA2 expression were correlated with higher grades of RCC and could be a risk factor for accelerated disease recurrence and indicative of a poor prognosis in surgically treated patients with RCC [6].

Focal adhesion kinase (FAK) regulates the dynamic of focal adhesion complexes–sites of attachment between cells and the extracellular matrix [7]. FAK plays prominent roles in malignant cellular behavior by regulating aspects of both cancer cells and their microenvironments such as cell migration, invasion, suppression of apoptosis and angiogenesis [7,8]. Previous in vitro studies using RCC cells have suggested potential roles of FAK in cancer development or progression [9–11]. Furthermore, a previous study showed that FAK was functionally important in EphA2 signaling and was a downstream effector in pancreatic adenocarcinoma cells [12]. Additionally, Rho GTPase proteins such as RhoA plays a role in cell survival/apoptosis, migration and invasion [13]. EphA can activate RhoA through FAK phosphorylation or exchange factors [14,15]. Recent studies have demonstrated that RhoA could act as an important signaling molecule that mediates EphA2 activation, promoting malignant cellular behavior in several types of cancer [16,17].

Based on these studies, it can be hypothesized that EphA2 plays critical roles in malignant cellular behavior such as resistance to apoptosis and invasiveness in human RCC cells. To date, there have been no studies that have investigated EphA2 expression or its role in malignant cellular behavior or the relationships among EphA2, FAK, and RhoA in RCC cells. Thus, the aim of this study was to determine the role of EphA2 in malignant cellular behavior of RCC cells and to identify whether FAK and RhoA can function as downstream effectors of EphA2 in various RCC cell lines using RNA interference mediated by small interfering RNA (siRNA).

## Materials and Methods

### Cell lines and Culture conditions

The human RCC cell lines (non-metastatic: Caki-2 and A498; metastatic: Caki-1 and ACHN), human embryonic kidney-293 (HEK-293) cells, and human prostate cancer cell lines (PC-3 and DU-145) were obtained from the American Type Culture Collection (Manassas, VA, USA). PC-3 and DU-145 were used as positive controls of EphA2 expression [18]. Each cell was incubated in Roswell Park Memorial Institute-1640 media containing 10% heat-inactivated fetal bovine serum and antibiotics (100 μg/mL of penicillin-streptomycin). Cells were grown as a monolayer on plastic cell culture dishes at 37°C in a humidified atmosphere containing 5% CO_2_.

### In vitro siRNA transfection

siRNA transfection of RCC cells was performed using Lipofectamine (Invitrogen, Carlsbad, CA, USA) according to the manufacturer’s instructions. The cells were seeded in a 6-well tissue culture plate at a density of 2 × 10^5^ cells/well and incubated at 37°C in a CO_2_ incubator for 24 hours. At that point, the cells were 50%–70% confluent. The cells were then transfected with control siRNA or siRNA for EphA2, FAK or RhoA for the indicated times. For each transfection, after combining diluted siRNA stock with 4 μL of Lipofectamine RNAiMAX reagent, the transfection complex was mixed gently and incubated for 20 minutes at room temperature. The transfection mixture was added to each well containing cells. The cells were then incubated at 37°C in a humidified CO_2_ incubator for the indicated times to assay for gene knockdown.

### Reverse transcription polymerase chain reaction (RT-PCR)

Total RNA was isolated from each cell using an RNeasy Mini Kit (Qiagen, Valencia, CA, USA) and TRIzol reagent (Invitrogen) according to the manufacturer’s recommended protocol. Aliquots of purified RNA (1 μg) was subjected to complementary DNA synthesis using an RT system (Promega, Madison, WI, USA) and oligo (dT). The total reaction volume was 20 μL. The primer sequences used for amplification were as follows: 5′-GGCTGTCGGTGTGGTCC-3′ as a sense primer and 5′-ATGTTGCGGGCAGCCAGGTC-3′ as an antisense primer for EphA2; 5′-CCACACCTTCTACAATGAGC-3′ as a sense primer and 5′-TGAGGTAGTCAGTCAGGTCC-3′ as an antisense primer for β-actin. PCR products were electrophoresed on 2% agarose gels and visualized using ethidium bromide staining and ultraviolet excitation.

### Western blot analysis

After control and transfected cells were incubated at 37°C in a CO_2_ incubator for the indicated times, total proteins were extracted from the cells. Additionally, the membrane fraction of RhoA was isolated by RhoA Western blot using a ProteoExtract Subcellular Proteome Extraction Kit Mini (Calbiochem, San Diego, CA, USA) using the manufacturer’s recommended protocol because the membrane fraction of RhoA contained most of the activated RhoA proteins. Equal amounts of the protein extracts were separated on SDS-polyacrylamide gels, transferred to polyvinylidene difluoride membranes, and incubated overnight with primary antibodies. The primary antibodies used were anti-EphA2 (1:1,000; Santa Cruz Biotechnology, Santa Cruz, CA, USA) or anti-FAK (1:2,000; Cell Signaling Technology, Danvers, MA, USA) or anti-phospho-FAK (Tyr397, 1:1,000, Cell Signaling Technology) or anti-RhoA (1:2000, Santa Cruz Biotechnology). The results were quantified by densitometry and normalized by β-actin expression.

### Cell viability assay

To identify whether the inhibition of EphA2 expression by using siRNA could inhibit proliferation of RCC cells, cellular viability was evaluated using a WST-8-based colorimetric assay in the Cell Counting Kit-8 (CCK-8, Dojindo Laboratories, Kumamoto, Japan) according to the manufacturer’s instructions. Cells were treated with siRNA for EphA2, control siRNA, and some cells were left untreated. The cells were incubated for 24–48 hours. After 24–48 hours of transfection, 10 μL of CCK-8 solution was added to 100 μL of medium in each well and the plates were incubated at 37°C in a CO_2_ incubator for 4 hours. The absorbance at 450 nm was measured using a VERSAmax microplate reader (Molecular Devices, Sunnyvale, CA, USA).

### Determination of the proportion of apoptotic cells

Control and transfected cells were harvested after 48 hours of transfection with siRNA for EphA2, FAK, RhoA, or control siRNA. An Annexin-V staining kit (Molecular Probes, Eugene, OR, USA) was used according to the manufacturer’s instructions to evaluate the proportions of apoptotic cells. The stained cells were analyzed by flow cytometry using a FACSCalibur flow cytometer (BD-Biosciences, San Jose, CA). Fluorescence emission was measured at 530 nm and 575 nm.

### Invasion assay

To investigate the role of EphA2/FAK/RhoA in cellular invasion of RCC cells, a modified Matrigel-Boyden chamber assay was performed at 48 hours post treatment with siRNA for EphA2, FAK, RhoA, or control siRNA. The BD BioCoat Matrigel invasion chamber (BD Bioscience, Bedford, MA, USA) was used according to the manufacturer’s protocol. After staining with Crystal Violet 1% solution, the number of cells that invaded through the Matrigel coated Transwell inserts were measured at 595 nm using a VERSAmax microplate reader (Molecular Devices).

### Three-Dimensional (3D) Culture Cell Invasion assay

To mimic cellular behavior in vivo and provide a more physiological approach for assessing it, we performed 3D culture cell invasion assay for RCC cells treated with EphA2 siRNA or control siRNA or untreated cells. The 96 Well 3D Spheroid BME Cell Invasion Assay kit (Catalog #3500-096-K, Amsbio, Abingdon, UK) was used according to the manufacturer’s protocol. The 3D spheroid images were obtained at the indicated times using the Leica Microsystems Welzlar GmbH microscope (type 090–135.001, Leica Microsystems, Wetzlar, Germany). Images were analyzed using the imageJ (Fiji).

### Statistical analysis

All data were obtained from at least three independent experiments. All variables were reported as mean ± standard error. The differences between groups were analyzed with the Mann-Whitney U-test, as indicated. The reported p-values were two-sided and a p-value of < 0.05 was considered statistically significant. SPSS version 12.0 (Chicago, IL, USA) was used for the analysis.

## Results

### Expression of EphA2 protein in RCC cells

**Fig 1:**
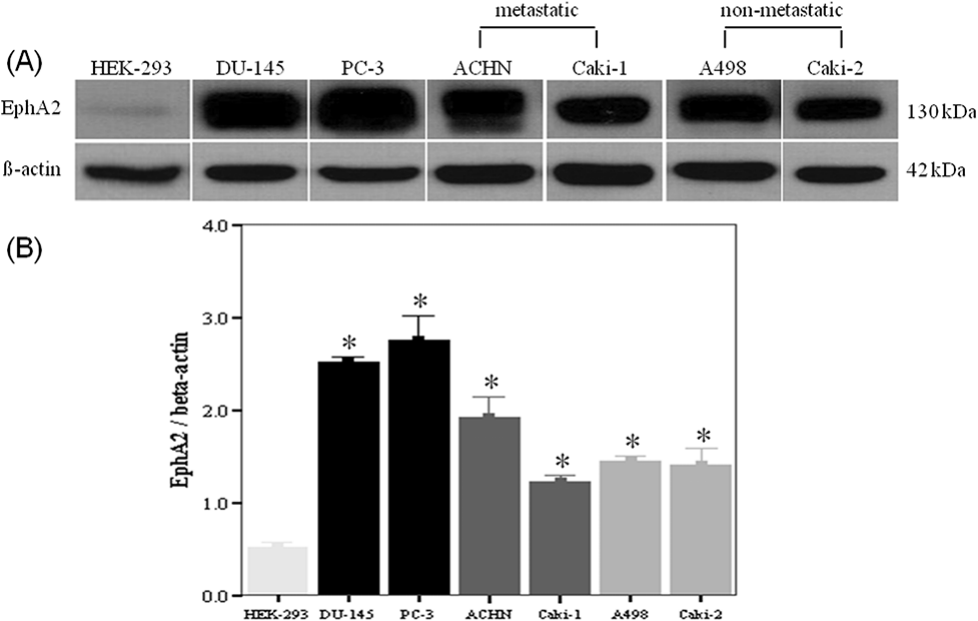
Western blot analysis demonstrating expression of EphA2 protein in human RCC cell lines. Representative immunoblots (A) and bar graphs (B) showing the comparison of EphA2 protein expression among the cell lines using densitometry. The results were normalized by β-actin expression. * p < 0.05 vs. HEK-293 cell. HEK-293 = human embryonic kidney-293, RCC = renal cell carcinoma.

To elucidate the role of EphA2 in the malignant cellular behavior of RCC cells, its expression was first confirmed in all cell lines by Western blot. In all RCC cell lines, the expression of EphA2 protein was detectable at variable levels while EphA2 expression in HEK-293 cell was very low (Fig 1).

### Effect of transfection with EphA2 siRNA on EphA2 expression in RCC cells

**Fig 2:**
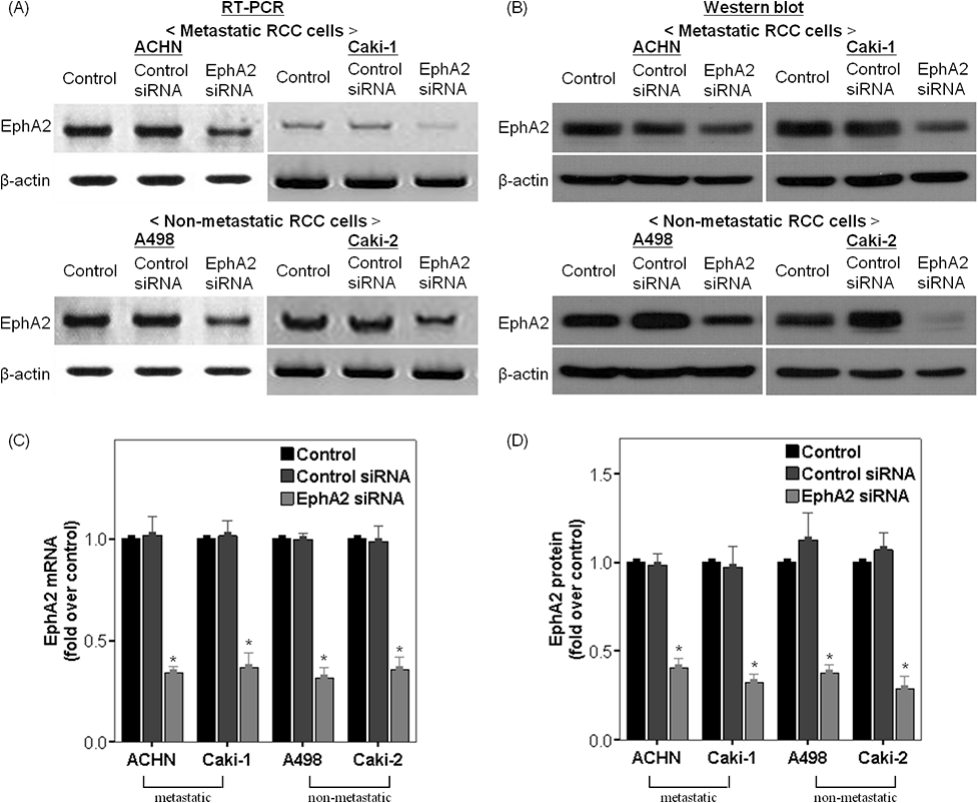
Effect of transfection with EphA2 siRNA on EphA2 expression in RCC cells. Representative RT-PCR (A) and corresponding immunoblots (B) for the comparison of EphA2 expression among the three groups in each cell line. The results were normalized by β-actin expression and presented as fold changes over controls (C and D). * p < 0.05 vs. control and control siRNA treated groups. siRNA = small interfering RNA, RT-PCR = Reverse transcription polymerase chain reaction, RCC = renal cell carcinoma.

The four RCC cell lines were treated with EphA2 siRNA or control siRNA. Cells were harvested at 48 hours post transfection and the expression of EphA2 was evaluated by RT-PCR and Western blot analysis (Fig 2). In all RCC cell lines, the expression of EphA2 proteins at 48 hours after EphA2 siRNA transfection was significantly lower than samples transfected with control siRNA and samples that were left untreated (Fig 2B and 2D). Similarly, EphA2 siRNA treatment suppressed EphA2 mRNA expression to varying degrees in all RCC cell lines (Fig 2A and 2C).

### Effect of EphA2 siRNA on cellular viability in RCC cells

**Fig 3:**
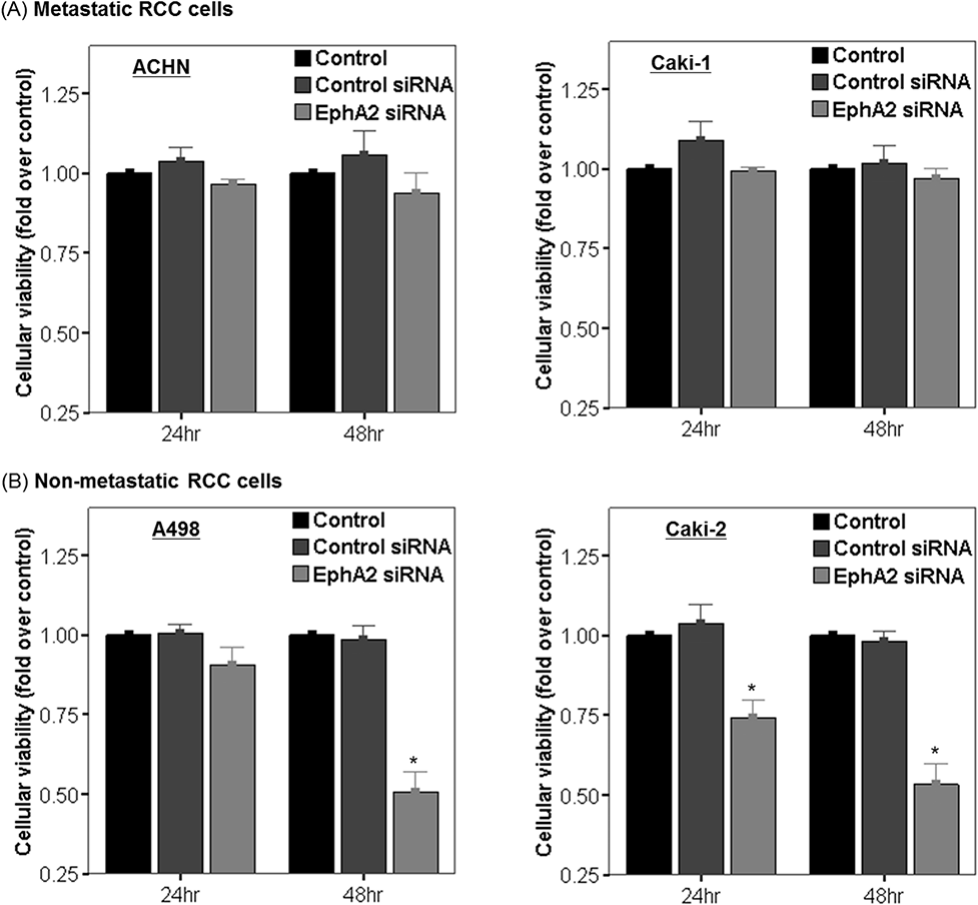
Effect of EphA2 siRNA on cellular viability in (A) metastatic RCC cell lines (ACHN and Caki-1) and (B) non-metastatic RCC cell lines (A498 and Caki-2). Cellular viability was compared among untreated cells (control), control siRNA treated cells and EphA2 siRNA treated cells at 24 and 48 hours following treatment in each cell line. The results were presented as fold changes over controls. * p < 0.05 vs. control and control siRNA treated groups. siRNA = small interfering RNA, RCC = renal cell carcinoma.

In the Caki-2 cell line (a non-metastatic RCC cell line), the viable cell count at 24 and 48 hours following EphA2 siRNA transfection was significantly decreased compared to untreated control or cells treated with control siRNA (Fig 3B). The cell viability in the A498 cell line (another non-metastatic RCC cell line) was significantly decreased at 48 hours following EphA2 siRNA transfection compared to untreated control or cells treated with control siRNA, but not at 24 hours after EphA2 siRNA treatment (Fig 3B). A slight decrease in cell viability was also observed in the metastatic RCC cell lines (Caki-1 and ACHN) treated with EphA2 siRNA. This was not significant compared to untreated control or cells treated with control siRNA (Fig 3A).

### Inhibition of EphA2 suppresses malignant behavior in non-metastatic RCC cells, but not in metastatic RCC cells

**Fig 4:**
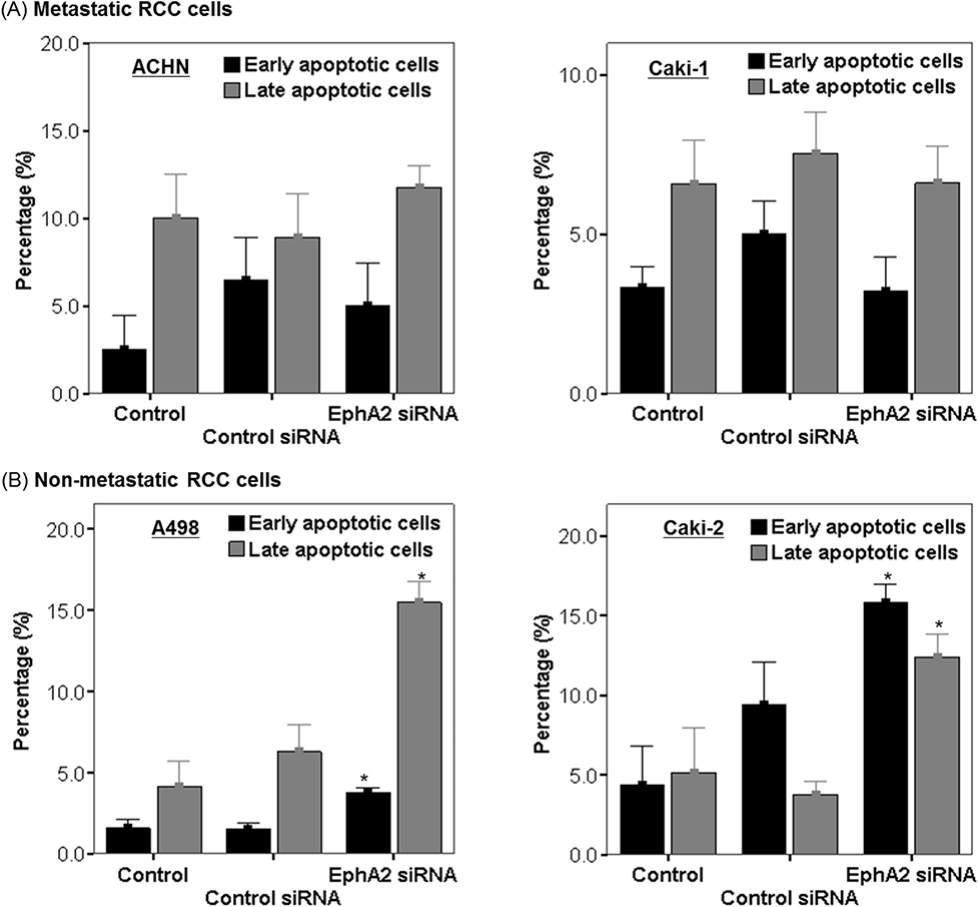
Evaluation of early or late apoptosis following EphA2 siRNA treatment in (A) metastatic RCC cell lines (ACHN and Caki-1) and (B) non-metastatic RCC cell lines (A498 and Caki-2). The proportion of apoptotic cells was compared among untreated cells (control), control siRNA treated cells and EphA2 siRNA treated cells at 48 hours following treatment in each cell line. * p < 0.05 vs. control and control siRNA treated groups. siRNA = small interfering RNA, RCC = renal cell carcinoma.

To clarify the role of EphA2 in the malignant behavior of RCC such as apoptosis resistance and cellular invasiveness, Annexin-V and modified Matrigel-Boyden chamber assays were performed, respectively. EphA2 siRNA treatment promoted early or late apoptosis at 48 hours following transfection in the non-metastatic RCC cell lines (Caki-2 and A498) (Fig 4B), but not in the metastatic RCC cell lines (Caki-1 or ACHN) (Fig 4A).

**Fig 5:**
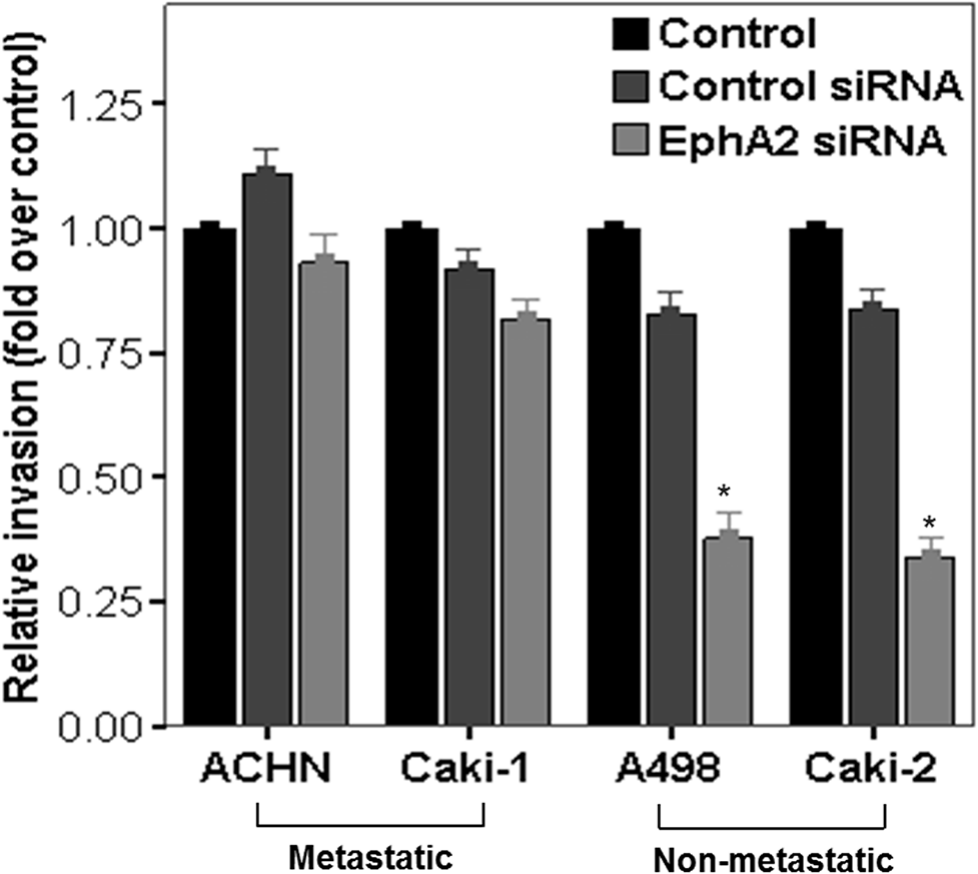
Effect of EphA2 siRNA on cellular invasiveness in human RCC cell lines. Cellular invasiveness was compared among untreated cells (control), control siRNA treated cells and EphA2 siRNA treated cells at 48 hours following treatment in metastatic RCC cell lines (ACHN and Caki-1) and non-metastatic RCC cell lines (A498 and Caki-2) Bar graph compared cellular invasiveness among the three groups using densitometry. The results were presented as fold changes over controls. * p < 0.05 vs. control and control siRNA treated groups. siRNA = small interfering RNA, RCC = renal cell carcinoma.

According to the modified Matrigel-Boyden chamber assay, cellular invasiveness of the non-metastatic RCC cell lines (Caki-2 and A498) was significantly decreased in EphA2 siRNA-treated cells after 48-hours of transfection compared to cells treated with control siRNA and the untreated control (Fig 5). However, EphA2 siRNA had no effect on cellular invasiveness in the metastatic RCC cell lines (Caki-1 or ACHN) (Fig 5).

**Fig 6:**
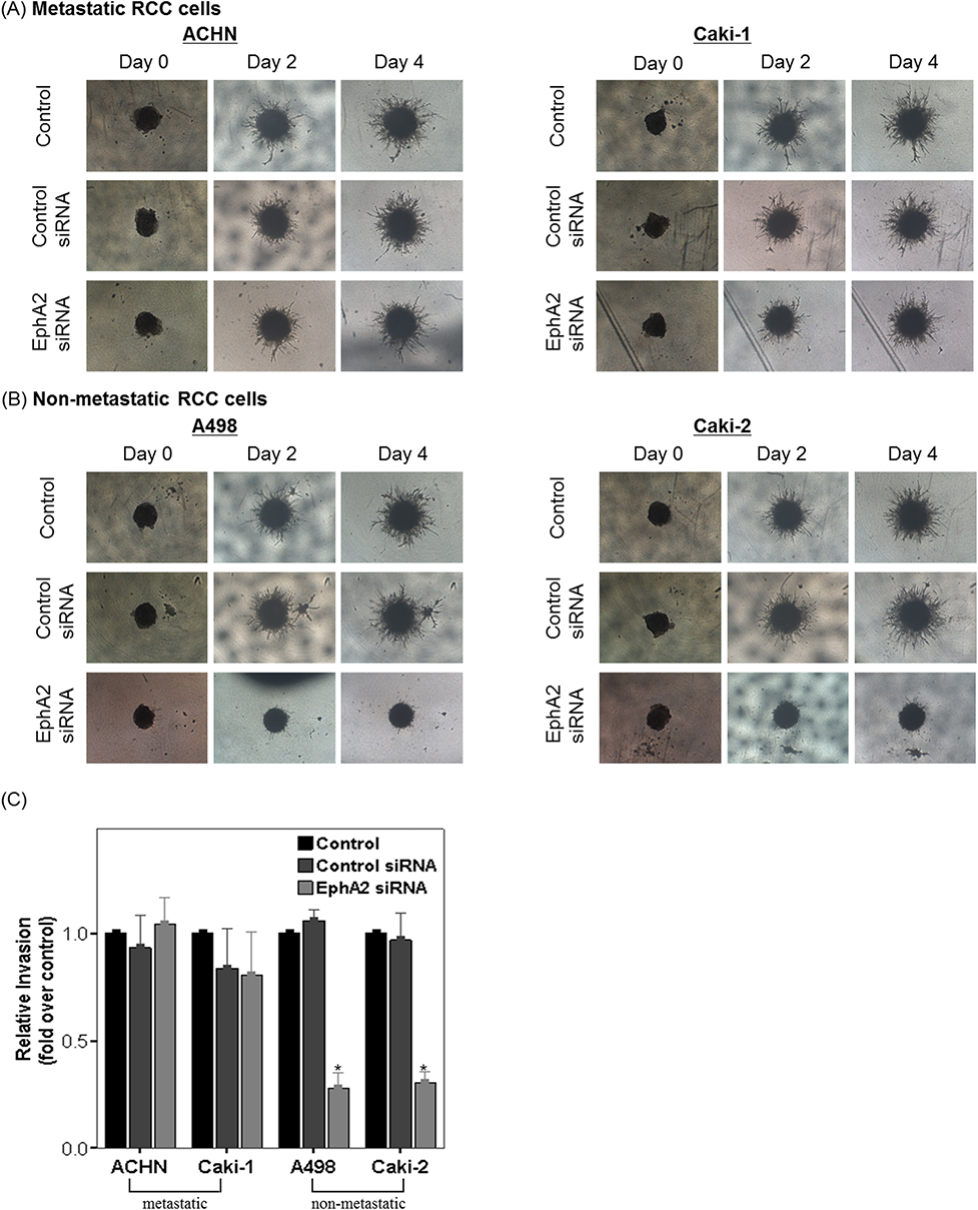
Effect of EphA2 siRNA on cellular invasiveness in 3-dimensional (3-D) cell culture models of human RCC cell lines. 3-D cell invasion was compared among untreated cells (control), control siRNA treated cells and EphA2 siRNA treated cells in each cell line. Representative images taken over a four-day period (A) metastatic RCC cell lines (ACHN and Caki-1) and (B) non-metastatic RCC cell lines (A498 and Caki-2). (C) Bar graph showing relative invasion in each cell line. Relative invasion was defined as a ratio: (the area obtained on day 4)/ (the area obtained on day 0). The results were presented as fold changes over controls. * p < 0.05 vs. control and control siRNA treated groups. siRNA = small interfering RNA, RCC = renal cell carcinoma

The results of 3D culture cell invasion assays were nearly identical to the corresponding results obtained with the modified Matrigel-Boyden chamber assays. EphA2 siRNA treatment suppressed the cellular invasion in the non-metastatic RCC cell lines (Caki-2 and A498) (Fig 6B and 6C), but not in the metastatic RCC cell lines (Caki-1 or ACHN) (Fig 6A and 6C).

### Effect of EphA2 siRNA on FAK phosphorylation and expression of membrane-bound RhoA in RCC cells

**Fig 7:**
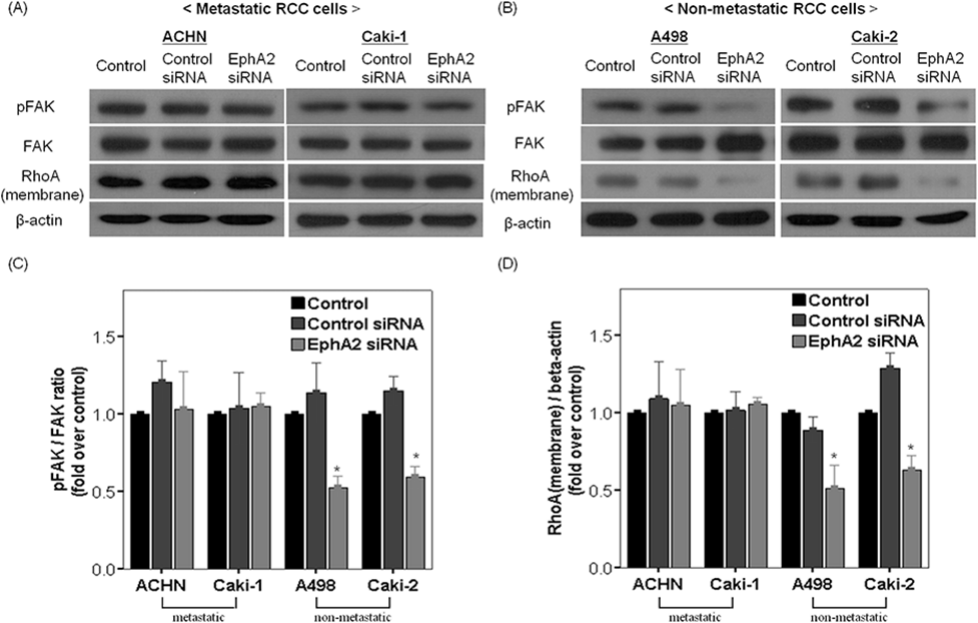
Effect of EphA2 siRNA on FAK phosphorylation and expression of membrane-bound RhoA in RCC cells. Representative immunoblots for pFAK, FAK, RhoA (membrane) and β-actin from untreated cells (control), control siRNA treated cells and EphA2 siRNA treated cells at 48 hours after treatment in (A) metastatic RCC cell lines (ACHN and Caki-1) and (B) non-metastatic RCC cell lines (A498 and Caki-2). Comparison of FAK phosphorylation (C) and the expression of RhoA (membrane) protein (D) among the three groups in each cell line. The results were normalized by β-actin expression and presented as fold changes over controls. * p < 0.05 vs. control and control siRNA treated groups. siRNA = small interfering RNA, RCC = renal cell carcinoma.

To clarify whether FAK or RhoA can function as a downstream effector of EphA2 in RCC cells, we examined the effect of EphA2 siRNA-mediated knockdown on FAK phosphorylation and expression of membrane-bound RhoA protein in all RCC cell lines (Fig 7). After 48 hours of EphA2 siRNA transfection in the non-metastatic RCC cell lines (Caki-2 and A498), FAK phosphorylation was decreased compared with cells treated with control siRNA or untreated control (Fig 7B and 7D). However, EphA2 siRNA had no effect on FAK phosphorylation in the metastatic RCC cell lines (Caki-1 or ACHN) (Fig 7A and 7C). At 48 hours after transfection with EphA2 siRNA, expression of membrane-bound RhoA followed the same trends as FAK phosphorylation (Fig 7). Treatment with EphA2 siRNA significantly reduced the expression of membrane-bound RhoA protein in the non-metastatic RCC cell lines (Caki-2 and A498) (Fig 7B and 7D) but not in the metastatic RCC cell lines (Caki-1 or ACHN) (Fig 7A and 7C).

### Malignant cellular behavior associated with increased expression of EphA2 is dependent on FAK/RhoA signaling in non-metastatic RCC cells

**Fig 8:**
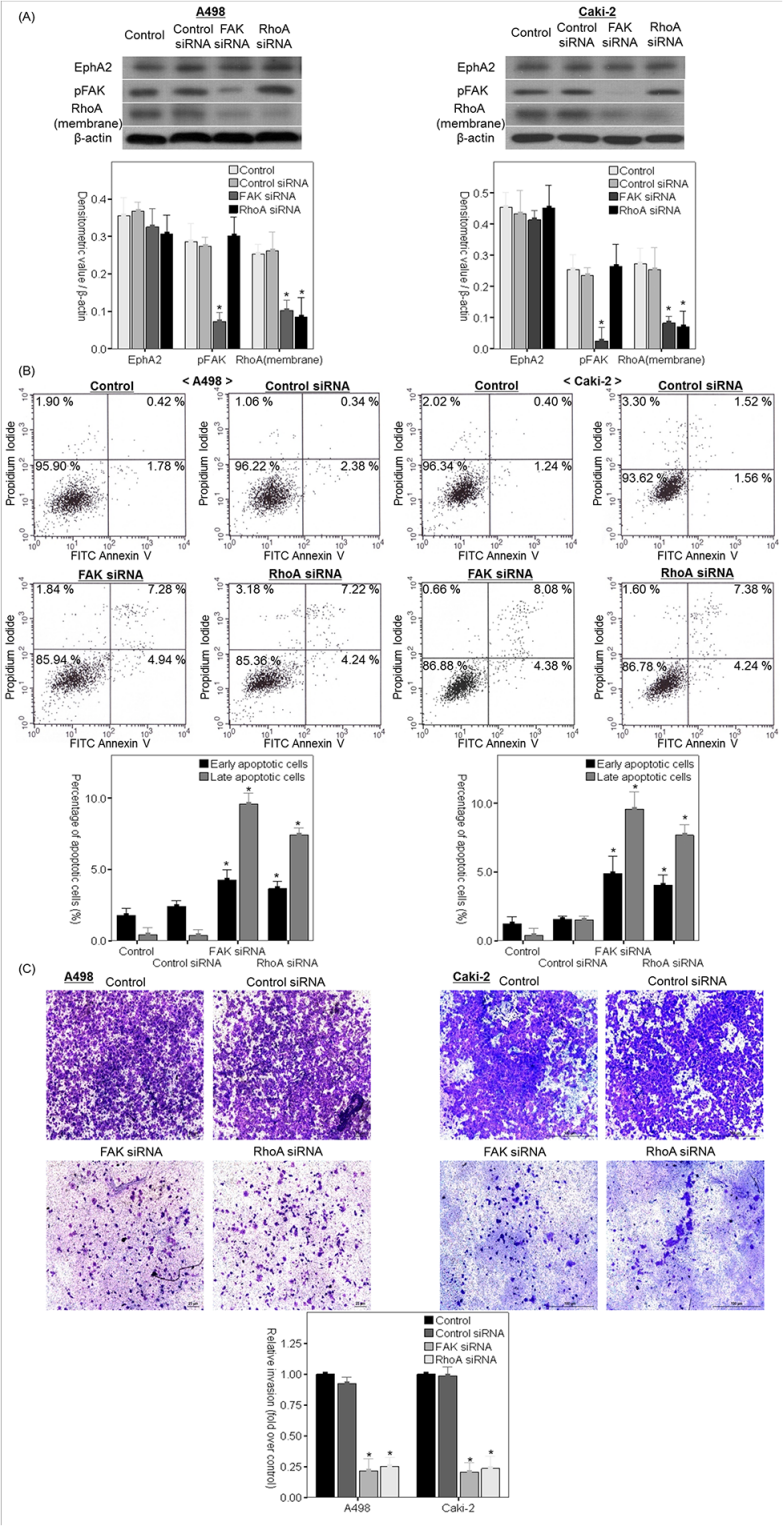
Effect of FAK/RhoA siRNA on malignant cellular behavior and EphA2 expression in non-metastatic RCC cells. (A) Representative immunoblots and comparisons of protein expression of EphA2, pFAK, membrane-bound RhoA and β-actin among untreated cell**s** (control), control siRNA treated cell**s** and FAK or RhoA siRNA treated cell**s** at 48 hours after treatment in each cell line. (B) Determination of the percentage of early or late apoptotic cells relative to the total number of cells following FAK or RhoA siRNA treatment in each cell line. (C) Effect of FAK and RhoA siRNA on cellular invasiveness in each cell line. * p < 0.05 vs. control and control siRNA treated groups. siRNA = small interfering RNA, FAK = focal adhesion kinase, pFAK = phosphorylated focal adhesion kinase, RCC = renal cell carcinoma.

To clarify whether malignant cellular behavior associated with increased expression of EphA2 was dependent on FAK or RhoA in the non-metastatic RCC cells, we examined the effects of FAK and RhoA siRNA-mediated knockdown on malignant cellular behavior in non-metastatic RCC cell lines (Caki-2 and A498) (Fig 8)

In the non-metastatic RCC cells (Caki-2 and A498), FAK siRNA treatment significantly promoted apoptosis (Fig 8B) while it significantly decreased cellular invasiveness and protein expression of membrane-bound RhoA without changing EphA2 protein expression at 48 hours following transfection (Fig 8A and 8C). Additionally, apoptosis was significantly enhanced (Fig 8B) and cellular invasiveness was significantly decreased (Fig 8C) without changes in expression of EphA2 protein or FAK phosphorylation (Fig 8A) after 48-hours of transfection in RhoA siRNA-treated cells compared to cells treated with control siRNA and the untreated control.

## Discussion

Tumor cells that overexpress EphA2 exhibit enhanced malignant cellular behavior such as resistance to apoptosis and cellular invasiveness [12,16,19,20]. Herrem et al. demonstrated that EphA2 expression levels might serve as a correlate for histologic grade and a prognostic indicator of cancer recurrence and survival in RCC patients treated with surgery [6]. However, the study evaluated only effects of expression level of EphA2 on survival or recurrence in surgically treated patients with RCC, using immunohistochemical analysis. It did not investigate the direct effects of EphA2 on the malignant cellular behavior of RCC cells. To our knowledge, this is the first report to characterize EphA2 expression and its contribution to malignant cellular behavior in various RCC cell lines. Additionally, given the critical roles of EphA2/FAK or RhoA signaling in malignant cellular behavior in various types of tumors, it is reasonable to investigate whether FAK or RhoA can act as a downstream effector of EphA2 in various RCC cell lines [12,16,17]. The major findings of our study can be summarized as follows: 1) EphA2 may play critical roles in malignant cellular behavior such as resistance to apoptosis and partially plays a role in cellular invasiveness in non-metastatic RCC cells but not in metastatic RCC cells; 2) FAK/RhoA signaling pathway can function as downstream effectors of EphA2 in non-metastatic RCC cells.

In the present study, EphA2 siRNA treatment attenuated cellular viability, resistance to apoptosis and invasiveness in non-metastatic RCC cell lines but not in metastatic RCC cells. The reason behind this finding is not entirely clear. EphA2 siRNA treatment reduced both survival and invasiveness, and the decreased viability or increased apoptosis could have partially contributed to the decreased invasion in the non-metastatic RCC cells. Based on this finding, increased EphA2 expression may play an important role in malignant cellular behavior in non-metastatic RCC cells. Specifically, increased EphA2 expression may enhance malignant behavior by decreasing apoptosis, and promoting viability and, in part, invasion of RCC cells in non-metastatic RCC. In accordance with our results, previous studies demonstrated that inhibition of EphA2 overexpression by siRNA knockdown or blocking antibody could lead to decreased malignant cellular behavior or progression in a variety of cancer cells [12,17,19–21].

One of the interesting findings revealed in this study is the observation that EphA2 siRNA treatment can significantly attenuate FAK phosphorylation and expression of membrane-bound RhoA in non-metastatic RCC cells but not in metastatic RCC cells, which is consistent with the corresponding results about viability, resistance to apoptosis and invasion after EphA2 siRNA transfection. A previous study demonstrated that FAK phosphorylation could play a critical role in resistance to apoptosis [22]. Duxberry et al. showed that EphA2 siRNA significantly suppressed FAK phosphorylation as well as resistance to apoptosis or cellular invasion in human pancreatic adenocarcinoma cells [12]. Thus, the level of EphA2 available for association with FAK can be decreased by inhibition of EphA2 expression with siRNA and the resulting decrease in FAK phosphorylation may contribute to suppression of resistance to apoptosis and partially contribute to invasiveness. Additionally, EphA can activate RhoA through FAK phosphorylation or exchange factors Ephexin and Vsm-RhoGEF [14,15]. Thus, EphA2 may play critical roles in resistance of apoptosis and invasion by activating Rho-GTPase which participates in survival/apoptosis. Recently, Brantley-Sieders et al. demonstrated that EphA2 could promote malignant cellular behavior through activation of RhoA in mammary adenocarcinoma cells [16]. Similarly, our study demonstrated that the expression of membrane-bound RhoA protein was down-regulated by EphA2 siRNA in non-metastatic RCC cells, suggesting that RhoA could be a downstream effector of EphA2. Furthermore, the present study demonstrated that FAK siRNA and RhoA siRNA attenuated the expression of membrane-bound RhoA and malignant cellular behavior in non-metastatic RCC cells without changing the expression of EphA2 protein and of EphA2 protein/phosphorylated FAK, respectively. This is consistent with the previous studies by Parri et al. showing that EphA2 overexpression could enhance malignant cellular behavior of melanoma cells or prostate cancer cells via activation of the FAK/RhoA signaling pathway [17,23]. Taken together, EphA2 might enhance malignant cellular behavior or progression of non-metastatic RCC cells through activation of FAK/RhoA signaling pathway.

A shortcoming of the present study is that we did not demonstrate our findings by using in vivo experiments in animal models. Alternately, we conducted a 3-dimentional cell culture model experiment in order to mimic cellular behavior in vivo and provide a more physiological approach for assessing it. Nevertheless, our results have an important clinical implication. EphA2/FAK/RhoA signaling may be important in malignant cellular behavior, particularly of non-metastatic RCC. Therefore, in the clinical setting, early therapeutic strategies targeting the above molecules may be helpful to prevent progression of non-metastatic RCC (e.g., in the setting of adjuvant therapy).

## Conclusions

Our study provides the first functional evidence that EphA2 may play critical roles in malignant cellular behavior such as resistance to apoptosis and, in part, invasion in RCC cells and that EphA2 appears to be functional particularly in non-metastatic RCC cells. Additionally, FAK/RhoA signaling can act as downstream effectors of EphA2 for promoting tumor progression in non-metastatic RCC cells. Thus, our data indicate that EphA2/FAK/RhoA signaling may be an important determinant in malignant cellular behavior, particularly in non-metastatic RCC cells.
